# Obscured by administrative data? Racial disparities in occupational injury

**DOI:** 10.5271/sjweh.3611

**Published:** 2016-12-12

**Authors:** Erika L Sabbath, Leslie I Boden, Jessica AR Williams, Dean Hashimoto, Karen Hopcia, Glorian Sorensen

**Affiliations:** 1 Boston College School of Social Work, Chestnut Hill, MA, USA.; 2 Boston University School of Public Health, Department of Environmental Health, Boston, MA, USA.; 3 University of Kansas School of Medicine, Kansas City, KS, USA.; 4 Partners HealthCare, Occupational Health Services, Boston, MA, USA.; 5 Dana-Farber Cancer Institute, Center for Community-based Research, Boston, MA, USA.; 6 Harvard T.H. Chan School of Public Health, Department of Social and Behavioral Sciences, Boston, MA, USA.

**Keywords:** administrative data, health disparity, health inequality, healthcare worker, injury data, minority worker, occupational health, occupational injury, race, racial disparity, workplace injury

## Abstract

**Objectives:**

Underreporting of occupational injuries is well documented, but underreporting patterns may vary by worker characteristics, obscuring disparities. We tested for racial and ethnic differences in injury reporting patterns by comparing injuries reported via research survey and administrative injury database in the same group of healthcare workers in the US.

**Methods:**

We used data from a cohort of 1568 hospital patient-care workers who were asked via survey whether they had been injured at work during the year prior (self-reported injury; N=244). Using the hospital’s injury database, we determined whether the same workers had reported injuries to the hospital’s occupational health service during that year (administratively reported injury; N=126). We compared data sources to test for racial and ethnic differences in injury reporting practices.

**Results:**

In logistic regression models adjusted for demographic and occupational characteristics, black workers’ odds of injury as measured by self-report data were 1.91 [95% confidence interval (95% CI) 1.04–3.49] compared with white workers. The same black workers’ odds of injury as measured by administrative data were 1.22 (95% CI 0.54–2.77) compared with white workers.

**Conclusions:**

The undercount of occupational injuries in administrative versus self-report data may be greater among black compared to white workers, leading to underestimates of racial disparities in workplace injury.

Minority and otherwise marginalized workers are dis-proportionately exposed to dangerous working conditions (1, 2). They may also be more likely to take low-paying or hazardous jobs because of undocumented legal status, chronic job insecurity, and social or educational barriers (3, 4). As a result, health outcomes associated with adverse working conditions often occur along social and economic gradients. Health disparities may be exacerbated by economic and labor policies that exclude vulnerable workers from workplace protections (5).

In the US, the overall rate of reported occupational injuries in 2014 was 3.4 per 100 full-time equivalent employees (FTE), with some industries – particularly healthcare, construction, and public safety – having >10 reported injuries per 100 FTE (6). Injury epidemiology is tracked through multiple administrative databases at different levels, including statewide and national occupational health surveillance systems, worker’s compensation databases, or nationally representative secondary datasets containing information on working conditions and health outcomes (7).

Underreporting and undercounting of injuries is well documented. By some estimates, up to 70% of occupational injuries are not captured in administrative databases (8, 9). An injury can get “lost” at several points in the reporting process: workers may be afraid to report injuries to supervisors; they may be unable to afford time away from work to recover; they may receive care outside of the workers’ compensation system; and injuries or illnesses may not be immediately recognizable as work-related (10). Nurses in hospital settings face several specific underreporting risks: time pressure, a professional ethos of putting others’ needs first, and fear of retaliation or resentment by peers (11).

Although occupational health disparities overall are well documented, barriers remain to understanding the true nature of disparities in injury reporting. Many studies of occupational injury use administrative data. These data cover a broad range of workers across industries, occupations, and settings, but they have shortcomings: optional (and thus incomplete) reporting of variables such as race (12); the need to create denominators from other datasets to obtain rates (7); and difficulty capturing workers who do not report injuries (2, 10). Furthermore, the extent of differential undercount by demographic group is unknown.

A recent study showed discrepancies between self-reported and administrative injury data among hospital patient-care workers (13). Our aim was to determine whether such discrepancies varied by racial and ethnic groups. Based on literature on barriers to injury reporting (7, 10, 11), it is possible that underrepresented minority workers may be less likely to use formal reporting processes when injured at work, but may reveal injuries when asked in a confidential survey. Thus, we hypothesized that we would detect greater racial and ethnic disparities in injury using self-reported rather than administrative data.

## Methods

### Sample

The study took place in a cohort of patient-care workers employed in two large hospitals (“Hospital A” and “Hospital B”) in the Boston area. In September 2012, 2000 workers were randomly sampled for the survey. Those eligible included staff nurses [registered nurses (RN) and clinical nurse specialists] and patient-care associates (PCA) employed by the hospitals for ≥20 hours per week. We excluded other healthcare professionals (eg, phlebotomists), administrative employees, those on leave for 12 weeks, and traveling or per diem nurses. Workers were retained if they completed >50% of the survey. Of the 2000 sampled workers, 1594 (79%) responded and were included. We eliminated participants missing data on self-reported race and ethnicity (N=15) or self-reported injury (N=11) for a final cohort of 1568.

### Outcome

Both self-report survey and administrative data were used to measure occupational injury. On the survey, respondents were asked, “During the past 12 months, including sharps injuries, were you injured seriously enough while performing your job that you got medical advice or treatment or lost time from work?” Response options were: “No, I did not have an injury” (coded “not injured”) “Yes, I had one injury and did report it (coded “injured and reported”), “Yes, I had one injury and did not report it” (coded “injured and unreported”), “Yes, I had more than one injury and did report all of them” (coded “injured and reported”), and “Yes, I had more than one injury and did report one but not all of them” (coded “injured and reported”).

To capture administratively reported injuries, we used data from the hospital’s administrative injury database (hereafter referred to as “administrative data” or “administratively reported injuries”) and merged those data with survey data at the worker level using secure study ID numbers. When a worker is injured, they are instructed to contact occupational health services (OHS), which enters the details of the injury and follows up with the worker until the case is closed. Alternatively, workers may complete a paper (Hospital B) or electronic (Hospital A) injury report form, which is then added to the administrative database. Here, we included all recordable injuries according to Occupational Health and Safety Administration (OSHA) rules (injuries resulting in medical treatment or time away from work) occurring in the 365 days prior to the individual worker’s completing the survey to maximize comparability between self-report and administrative injury data. More minor injuries are included in the database, but we used only OSHA-recordable injuries to capture the same construct as was measured in the survey. If workers administratively reported more than one injury during the year prior, we categorized them as injured if ≥1 of those injuries was OSHA recordable.

We cross-tabulated self-report and administrative data for each worker and categorized each worker by both their self-reported injury status and administratively reported injury status, for a total of four categories.

### Exposure

In the survey, workers were asked, “Do you consider yourself Latino or Hispanic?” (response options: “Yes: includes Puerto Rican, Cuban American, Mexican American, etc.” and “No, not Latino/Hispanic or Spanish.”). They were then asked “How would you describe your race? Please check all that apply” (response options: Native American or Alaska native; Asian, Asian American; Native Hawaiian or Pacific Islander; black, African-American; white; and other). We recoded the preceding variables into a single race/ethnicity variable with categories Hispanic; non-Hispanic white; non-Hispanic black; mixed race/other. The small number of minority workers prevented us from including more categories of race and ethnicity in analyses. Throughout this manuscript, “black workers” refers to non-Hispanic black workers.

### Covariates

We included gender (male, female); job title (nurse, PCA, other); immigrant status (native- versus foreign-born), typical shifts worked (days, evenings, nights, rotating), age (≤30, 31–40, 41–50, ≥51 years), personal financial distress (14), and hospital site at which the person worked (A or B). All covariates were assessed in the survey.

### Analyses

We first examined distribution of covariates overall and by race/ethnicity. We then tabulated frequencies of self-reported occupational injury (categories: not injured, injured and said that they reported it, injured and said that they did not report it), administratively reported injury (did not report any injuries; reported ≥1 injury), and concordance between self-reported and administratively reported injuries, overall and by race/ethnicity. We used logistic regression to model two different binary outcomes – self- and administratively reported injury – adjusting progressively for demographic/personal and then occupational covariates.

We conducted sensitivity analyses using administrative injuries occurring up to 15 months before the survey (to capture workers’ misremembering injury timing) and all administratively reported injuries (not just OSHA-recordable) during the 12 prior months.

As the mixed race/other race or ethnicity category was quite heterogeneous, we have included this group in tables but do not discuss results at length.

### Human subjects

The human subjects committee at the Harvard TH Chan School of Public Health approved this study (protocol #22228).

## Results

Of the total sample, approximately 81% (N=1266) identified as non-Hispanic white, 4% (N=64) as Hispanic, 8% (N=131) as non-Hispanic black, and 7% (N=107) as mixed-race or other (table 1). The overall sample was 93% women, 85% staff nurses, and 85% native-born. Workers most commonly had rotating shifts (43%) and were evenly distributed across 10-year age groups; 15% were financially distressed.

We found substantial racial/ethnic variation (P for between-category differences <0.0001) in financial distress, immigrant status, job title, and hospital site. Among white workers, 92% were staff nurses and 4% were PCA (a low-wage job). Among Hispanic workers, 39% were staff nurses and 39% were PCA; among black workers, 42% were staff nurses and 50% were PCA. Of the foreign-born workers, 4%, 60%, and 78% were white, Hispanic, and black, respectively, while 10% of white, 39% of Hispanic, and 55% of black workers were financially distressed. White workers were nearly twice as likely to work at Hospital A versus Hospital B.

We examined bivariate racial/ethnic differences in injuries. Overall, 16% of workers reported on the survey that they were injured during the year prior; among the injured, 73% said that they formally reported the injury and 27% did not. Both overall self-reported injury rate and self-admitted reporting practices varied by race and ethnicity. On the survey, 14% of white workers reported being injured; among the injured, 75% said that they reported the injury and 25% did not. Of the 20% of Hispanic workers who reported being injured, 69% said that they reported the injury and 31% did not. Of the 27% of black workers who reported being injured, 66% said that they reported the injury and 34% did not (χ^2^ P-value for racial/ethnic differences=0.003).

In parallel, we examined injury patterns using administratively reported injuries from the database. Based on these data, the overall injury rate was 8%. When examined by racial/ethnic group, 7% of white workers, 11% of Hispanic workers, and 12% of black workers reported injuries to OHS. These racial and ethnic differences in injury were not statistically significant (χ^2^ P=0.227).

**Figure 1.**
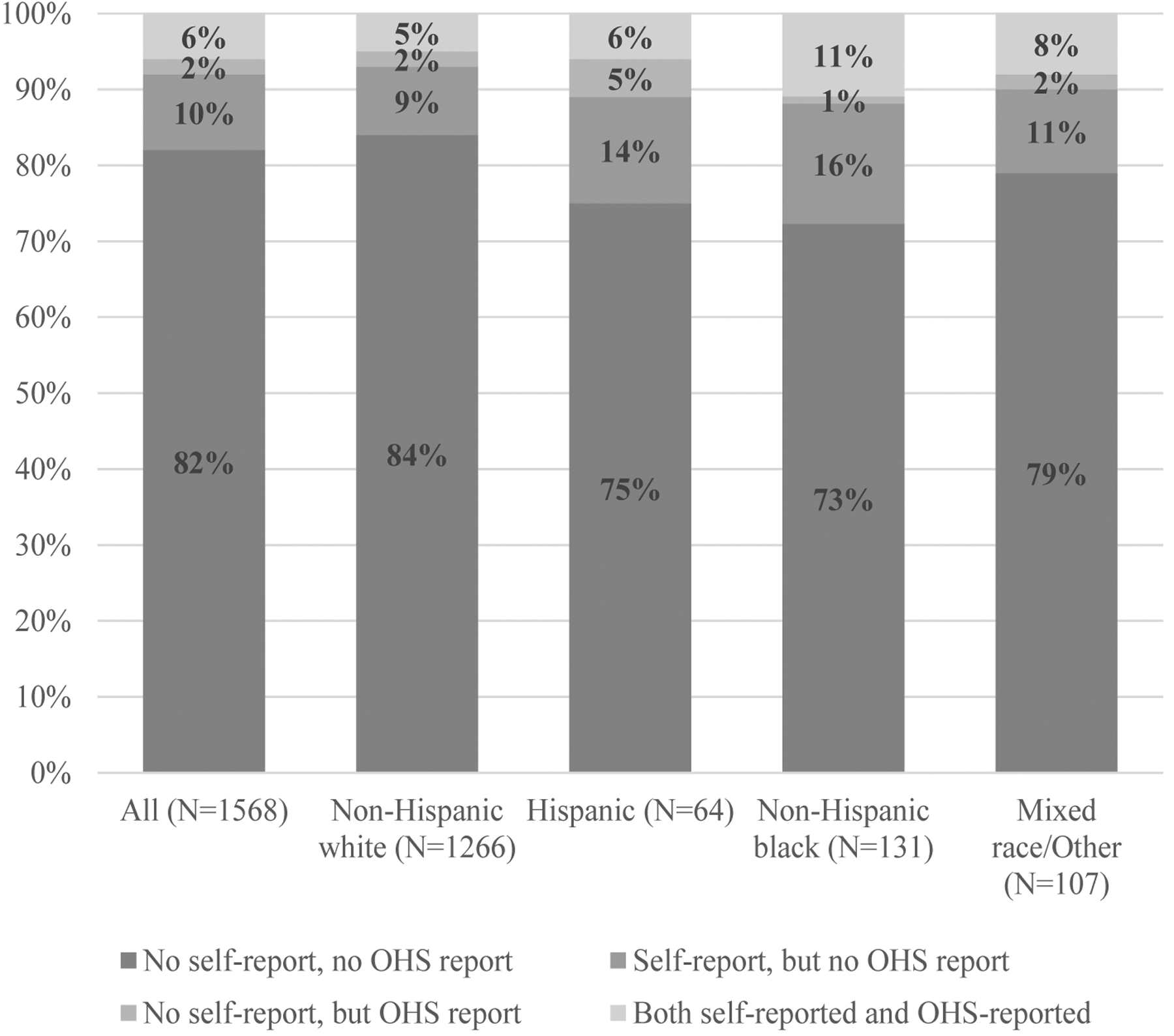
Concordance between self-reported occupational injury and administratively-reported reported injuries over the same one-year period, overall and by race/ethnicity. Chi-square P-value for difference in concordance between racial/ethnic groups=0.0099.

We cross-tabulated self-reports and administrative data (figure 1). Overall, 82% of workers had no injury by either source, 10% had a self-report but no administratively reported injury, 2% had an administratively reported injury but no self-report, and 6% had both self-and administratively reported injuries. Concordance again varied by racial and ethnic groups. Administrative underreporting – in which a worker self-reported an injury but did not appear in the administrative data – was 9% for white workers, 14% for Hispanic workers, and 16% for black workers (χ^2^ P-value for difference in injury reporting concordance by race/ethnicity=0.0099).

In unadjusted logistic regression models of associations between race/ethnicity and any self-reported injury on the survey (table 2), compared with white workers, black workers had an OR for injury of 2.27 (95% CI 1.50–3.46); risk was not significantly elevated for Hispanic workers (OR 1.59, 95% CI 0.85–3.46). In unadjusted models of associations between race/ethnicity and any administratively reported injury (table 3), compared with white workers, risks were not significantly elevated for either black (OR 1.63, 95% CI 0.92–2.91) or Hispanic (OR 1.55, 95% CI 0.69–3.49) workers.

Given strong covariation of race/ethnicity with individual characteristics factors in table 1, we first adjusted for age, gender, nativity, and financial distress. In these models, for self-reported injury, compared with white workers, Hispanic workers had a non-significant OR of 1.37 (95% CI 0.66–2.83) and black workers had a slightly attenuated, but still statistically significant, OR of 1.93 (95% CI 1.07–3.45). Using administratively reported injury with the same covariates, again neither black (OR 1.65, 95% CI 0.76–3.61) nor Hispanic (OR 1.59, 95% CI 0.64–3.96) workers had a statistically significantly elevated risk of injury.

In the final models, we adjusted for the preceding factors plus job title (also strongly associated with race/ethnicity in table 1), shift, and hospital site. For self-reported injury, black workers still had elevated risk of injury compared with white workers (OR 1.91, 95% CI 1.04–3.49), but Hispanic workers did not (OR 1.27, 95% CI 0.60–2.72). In the final model for administratively reported injury, neither black (OR 1.22, 95% CI 0.54–2.77) not Hispanic (OR 1.01, 95% CI 0.39–2.66) workers were at significantly greater risk than white workers.

We conducted sensitivity analyses to check the robustness of our findings (full results available upon request). In models using administratively reported injuries up to 15 months prior to the survey (instead of 12 months as in the main models), fully adjusted OR for black workers (versus white) was 1.18 (95% CI 0.53–2.64). Using all past-year administratively reported injuries (not just OSHA-recordable injuries), OR for black workers (versus white) was 0.98 (95% CI 0.52–1.82).

## Discussion

Compared with white workers, black workers had significantly increased risk of injury when asked via self-report but not when using administrative data. Undercounting of injuries in employers’ administrative data (on which national injury data are based) may be higher among black workers, obscuring racial disparities in risk.

This study raises two questions. First, why do administrative and self-report data present different pictures of racial disparities in occupational injury? Especially given nativity differences by race in our sample (table 1), immigration status is a natural explanation: foreign-born workers may not be aware of injury reporting practices in the US or may fear visa loss or deportation (10). But after adjusting for immigration, racial differences remained, and nativity was not a significant predictor of injury in the presence of race.

Financial distress was more common among black and Hispanic than among white workers in our sample (χ^2^ P<0.0001) and could theoretically reduce willingness to report an injury (reporting could result in determination that injured workers would need to miss work to recover). But race was significantly associated with self-reported injury rates even in the presence of financial distress – itself an independent predictor of self-reported injury – and financial distress was not associated with administratively reported injuries. Thus, it failed to explain racial disparities in reporting.

Other explanations are more speculative. Historically, black individuals have had lower trust in healthcare systems and more suspicion of purported medical privacy (15, 16). If black workers feel that injury reports will not be kept confidential, willingness to report might be lower. The pattern could also be explained by “John Henryism”– a stress coping mechanism in which professional success is pursued, at the expense of one’s own health, to avoid conforming to negative racial stereotypes (17). In this scenario, injured black workers would choose to not report injuries to avoid impressions of malingering or inability to do the job competently.

The second question raised by this study is why, when reporting in a confidential survey versus an administrative report, do black workers have higher injury rates than white workers? Our initial explanation was differences in job types and functions between black and white workers – here, black workers were more likely to do low-wage jobs, which have higher risk for injury in healthcare settings, and have less power (18). However, when we adjusted for job type in table 3, Model 3, not only did racial disparities remain, but in the presence of race, job type was not a significant predictor of injury risk.

Health disparities literature posits that repeated experiences of discrimination can exert wear and tear on the body through stress pathways (19, 20), increasing risk of cardiovascular disease and its antecedents (21–23). Either overt or subtle discrimination at work could produce muscular tension as a physiological stress response, increasing risk of chronic musculoskeletal disorders over time because tense muscles may be more injury prone (24, 25). Black workers are at particular risk of being injured through this pathway because they may accumulate the physical toll of discrimination both within and outside of the workplace. Release of cortisol during an episode of discrimination could also lead to dulled pain perception and thus overexertion-related injuries (26), especially for workers with jobs requiring extensive lifting (most patient-care worker jobs).

The greatest limitation of this analysis is sample size. With 1568 workers, 302 of whom self-identified as non-minority (a category further divided among several racial and ethnic groups), cell sizes were small. This is a particular concern for the 64 Hispanic workers who had 13 and 7 self- and administratively reported injuries, respectively, dividing injuries into further subgroups and raising the risk of a Type II error. Sample size also precluded testing whether, in regression models, concordance between self- and OHS-reported injuries varied by race and ethnicity. The data only represent one employer and are relatively homogeneous by gender in addition to by race, potentially limiting generalizability. Finally, self-reported injury rate may be an underestimate if workers misremember timing or severity of their injuries, although we attempted to address this with a sensitivity analysis of injuries occurring up to 15 months pre-survey.

That said, this paper permits comparison, among individual workers and within a defined time period, of administratively versus self-reported injuries. We obtained self-reported race and ethnicity for all workers in the sample, a barrier to studying disparities in traditional administrative data because reporting of race is optional (7, 27). We adjusted for worker characteristics not available in administrative datasets, ruling out some alternative explanations.

Hospitals need to reduce barriers to injury reporting because administrative injury data is used for surveillance and policy-making at both the institutional and state levels. If such data undercount minority injuries, as occurred here, institutions will not recognize, and thus cannot address, racial disparities in injury. Better reporting for minority groups specifically could be accomplished through alternative or augmented data collection methods and better injury follow-up for vulnerable or chronically undercounted groups of workers (28). More sensitive data collection by employers and large population-based surveys, including routine collection of data on worker demographics such as race (10), could help reduce obscuring of disparities. Finally, if mistrust of reporting systems in minority or otherwise vulnerable communities is a consistent barrier to reporting, surveys conducted by trusted community partners could help improve reporting (29).

Workplaces should also address the underlying reasons why minority – particularly black – workers are injured at higher rates than white workers. This is particularly challenging in hospitals because experiences of discrimination may arise from patients’ mistreatment of workers rather than from colleagues over whom the hospital has more control (30). Because injury risk is lower in workgroups with greater cohesion and coworker support (31, 32), programs to improve psychosocial work environments could buffer minority workers from injury-related consequences of patient behavior.

## Figures and Tables

**Table 1.** Social, demographic, and occupational characteristics of sample, overall and by race/ethnicity.

	All (N=1568)		Non-Hispanic white (N=1266)		Hispanic (N=64)		Non-Hispanic black (N=131)		Mixed race/other (N=107)		X^2^ P-value
	N	%	N	%	N	%	N	%	N	%	
Gender											0.0123
Male	111	7.11	79	6.26	7	11.11	10	7.63	15	14.15	
Female	1450	92.89	1182	93.74	56	88.89	121	92.37	91	85.85	
Age											0.0019
≤30	456	29.08	376	29.7	25	39.06	20	15.27	35	32.71	
31–40	385	24.55	303	23.93	16	25	39	29.77	27	25.23	
41–50	336	21.43	258	20.38	16	25	41	31.3	21	19.63	
≥51	391	24.94	329	25.99	7	10.94	31	23.66	24	22.43	
Financial distress											<0.0001
No	1319	85.15	1134	90.07	39	60.94	56	45.16	90	88.24	
Yes	230	14.85	125	9.93	25	39.06	68	54.84	12	11.76	
Immigrant status											<0.0001
Native-born	1321	84.84	1214	96.12	26	40.63	29	22.48	52	51.49	
Foreign-born	236	15.16	49	3.88	38	59.38	100	77.52	49	48.51	
Job title											<0.0001
Staff nurse	1330	85.04	1162	92.08	25	39.06	55	41.98	88	82.24	
Patient care associate	137	8.76	33	2.61	25	39.06	65	49.62	14	13.08	
Other	97	6.2	67	5.31	14	21.88	11	8.4	5	4.67	
Typical shifts worked											0.0002
Days	412	26.34	326	25.77	26	41.27	31	23.85	29	27.36	
Evenings	86	5.5	57	4.51	6	9.52	17	13.08	6	5.66	
Nights	392	25.06	320	25.3	10	15.87	39	30	23	21.7	
Rotating shifts	674	43.09	562	44.43	21	33.33	43	33.08	48	45.28	
Hospital site											<0.0001
Hospital A	1003	63.97	836	66.03	31	48.44	61	46.56	75	70.09	
Hospital B	565	36.03	430	33.97	33	51.56	70	53.44	32	29.91	
Self-reported injury											0.0028
Was not injured	1324	84.44	1091	86.18	51	79.69	96	73.28	86	80.37	
Injured and reported	179	11.42	131	10.35	9	14.06	23	17.56	16	14.95	
Injured and did not report	65	4.15	44	3.48	4	6.25	12	9.16	5	4.67	
OSHA-reportable database injuries											0.2269
Did not report injury	1442	91.96	1173	92.65	57	89.06	116	88.55	96	89.72	
Reported ≥1 injury	126	8.04	93	7.35	7	10.94	15	11.45	11	10.28	

[OSHA=Occupational Safety & Health Administration.]

**Table 2.** Logistic regression modeling determinants of self-reported injury severe enough to require medical attention or miss work.

	Model 1		Model 2		Model 3	
	OR	95% CI	OR	95% CI	OR	95% CI
Race/ethnicity						
Non-Hispanic white	1.00		1.00		1.00	
Hispanic	1.59	0.85–2.98	1.37	0.66–2.83	1.27	0.60–2.72
Non-Hispanic black	2.27	1.50–3.46	1.93	1.07–3.45	1.91	1.04–3.49
Mixed race/other	1.52	0.92–2.52	1.40	0.77–2.53	1.42	0.78–2.59
Gender						
Male			1.00		1.00	
Female			0.96	0.56–1.64	0.90	0.52–1.56
Age (years)						
≤30			1.00		1.00	
31–40			1.21	0.80–1.82	1.23	0.80–1.87
41–50			1.31	0.86–2.01	1.31	0.84–2.04
≥51			1.96	1.33–2.89	1.96	1.30–2.96
Financial distress						
No			1.00		1.00	
Yes			1.65	1.12–2.42	1.67	1.11–2.51
Immigrant status						
Native-born			1.00		1.00	
Foreign-born			1.02	0.61–1.69	1.02	0.60–1.72
Job title						
Staff nurse					1.00	
Patient care associate					0.96	0.53–1.75
Other					1.03	0.58–1.85
Typical shifts worked						
Days					1.00	
Evenings					0.60	0.30–1.23
Nights					0.84	0.56–1.26
Rotating					1.16	0.81–1.68
Hospital site						
Hospital A					1.00	
Hospital B					1.77	1.32–2.39

[OR=odds ratio; 95% CI=95% confidence interval.]

**Table 3.** Logistic regression modeling determinants of administratively-reported (Occupational Safety & Health Administration (OSHA)-reportable injury.

	Model 1		Model 2		Model 3	
	OR	95% CI	OR	95%	OR	95% CI
Race/ethnicity						
Non-Hispanic white	1.00		1.00		1.00	
Hispanic	1.55	0.69–3.49	1.59	0.64–3.96	1.01	0.39–2.66
Non-Hispanic black	1.63	0.92–2.91	1.65	0.76–3.61	1.22	0.54–2.77
Mixed race/other	1.45	0.75–2.79	1.43	0.66–3.12	1.51	0.68–3.36
Gender						
Male			1.00		1.00	
Female			1.91	0.76–4.82	2.11	0.82–5.42
Age (years)						
≤30			1.00		1.00	
31–40			1.00	0.60–1.68	0.95	0.56–1.62
41–50			0.91	0.52–1.58	0.84	0.47–1.49
≥51			1.22	0.74–2.01	0.97	0.57–1.66
Financial distress						
No			1.00		1.00	
Yes			1.55	0.94–2.57	1.30	0.76–2.24
Immigrant status						
Native-born			1.00		1.00	
Foreign-born			0.85	0.43–1.69	0.73	0.35–1.51
Job title						
Staff nurse					1.00	
Patient care associate					1.72	0.80–3.70
Other					2.68	1.44–4.98
Typical shifts worked						
Days					1.00	
Evenings					1.19	0.55–2.56
Nights					0.84	0.50–1.43
Rotating					0.99	0.60–1.62
Hospital site						
Hospital A					1.00	
Hospital B					2.52	1.70–3.75

[OR=odds ratio; 95% CI=95% confidence interval.]
